# Self-reported dizziness, falls, and self-rated health in a rural population in Denmark

**DOI:** 10.1007/s00405-023-08061-2

**Published:** 2023-07-08

**Authors:** Casper Grønlund, Bjarki Ditlev Djurhuus, Ellen Astrid Holm, Preben Homøe

**Affiliations:** 1 Department of Otorhinolaryngology, Zealand University Hospital, Lykkebækvej 1, 4600 Køge, Denmark; 2 Department of Medicine, Zealand University Hospital, Køge, Denmark

**Keywords:** Health study, Lolland-Falster, Dizziness, Hearing loss, Cross-sectional, Falls

## Abstract

**Purpose:**

To investigate associations between dizziness, hearing loss, medication, and self-perceived health in the region of Lolland-Falster in Denmark.

**Methods:**

A cross-sectional population-based study using data from questionnaires and physical examinations between February 8th, 2016, and February 13th, 2020. Individuals aged 50 years or above in the region of Lolland-Falster were randomly invited to participate.

**Results:**

Of 10,092 individuals (52% female), the mean age was 64.7 and 65.7 years for females and males, respectively. 20% reported dizziness during the past 30 days, and prevalence increased with age. 24% of dizzy females suffered from falls compared to 21% of males. 43% sought treatment for dizziness. Logistic regression revealed a higher odds ratio of dizziness in groups with poor self-perceived health (OR = 2.15, 95% CI [1.71, 2.72]) and very poor self-perceived health (OR = 3.62 [1.75, 7.93]) compared to moderate self-perceived health. A higher OR was found for seeking treatment for dizziness in the group that had experienced falls (OR = 3.21 [2.54, 4.07]). 40% reported hearing loss. Logistic regression revealed a higher OR for dizziness in the group with severe hearing loss (OR = 2.40 [1.77, 3.26]) and moderate hearing loss (OR = 1.63 [1.37, 1.94]) compared to no hearing loss.

**Conclusion:**

One of five participants reported dizziness during the last month. Dizziness was negatively associated with self-perception of good health also after adjusting for comorbidities. Almost half of the dizzy participants sought treatment for dizziness and 21% experienced falls. Identification and treatment of dizziness are important to prevent falls from happening.

**Clinical trial registration:**

http://www.clinicaltrials.gov (NCT02482896).

## Introduction

Dizziness and vertigo are among the most common complaints in the healthcare system, affecting an estimated 15–35% of the population at some point in life [1]. Dizziness as a symptom is non-specific and may arise from several organ systems. Some of the most frequent reasons are vestibular, neurological, and cardiovascular disorders. Furthermore, dizziness often arises due to the side effects of medication.

In 2008, a national health interview survey found that 24.2 million adults in America (corresponding to 11.1% of the included individuals) had experienced dizziness during the past 12 months [2]. Among participants aged 65 and above, 19.6% reported a problem with dizziness or unsteadiness in the preceding 12 months [3], and women were more often affected than men (21% vs. 18%). These results have been confirmed by other similar studies [4, 5]. Concerning the quality of life and functional status, 37.3 million elderly individuals corresponding to 27.4% reported that balance problems prevented them from participating in activities such as social events, exercise, and driving [3]. This was also confirmed by a cohort study with 2751 participants reporting that adults affected by dizziness/vertigo generally had lower quality of life scores [6].

The Lolland-Falster Health Study (LOFUS) is a household-based population study based on people living in the two Danish islands of Lolland and Falster. Approximately 103,000 inhabitants live in this region, which is considered one of the most socio-economically disadvantaged areas in Denmark [7]. An analysis of the use of doctors, use of medication, and life expectancy, done in 2010 by the Workers Union, showed that the region has a higher occurrence of sickness and a lower average life expectancy than the rest of Denmark [8].

LOFUS was initiated to gain knowledge on determinants of health in a socio-economically disadvantaged and mainly rural area of Denmark using questionnaires, physical examinations, and biological samples hypothesizing that lifestyle, environment, and heritage together are risk factors for disease [7]. Other similar studies but in highly urbanized areas have been performed in Denmark [9].

In this report, we focus on dizziness, and hearing loss reported by the elderly participants in the LOFUS cohort. We would like to specify that we use dizziness as an umbrella term including vertigo and unsteadiness, as the Danish language does not distinguish between these. We hypothesize that these symptoms are frequent and are associated with poorer health perception and comorbidities. Also, we explore the use of doctors for these complaints.

## Methods

### Study design

From February 8th, 2016, to February 13th, 2020, 19,000 participants aged 0–96, participated in LOFUS. Participants answered questions concerning both ear-, nose-, and throat problems and socioeconomic status.

### Invitation, booking procedure, and visit

Individuals aged 18 years or above in the region of Lolland-Falster were randomly invited to participate in the Health Study. Invited individuals who agreed to participate were booked for an appointment in one of three static sites. The visit included a dialogue concerning written informed consent, followed by a physical examination. Thereafter, participants received a confirmation letter including a link and password to a web-based questionnaire [7].

### Eligibility criteria

The focus of the present study was dizziness. Dizziness was used as an umbrella term including vertigo and unsteadiness, as the Danish language does not distinguish between these. Inclusion criteria were age above 50 years and having answered the sub-questionnaire on dizziness. Exclusion criteria were incapacitated people with guardians, individuals without a permanent residence, inhabitants with address protection, and inhabitants unable to understand Danish or English [7].

### Sample population

The sample population was drawn from the target population of around 103,000 inhabitants in the region of Lolland-Falster from the two municipalities Lolland or Guldborgsund [7].

### Questionnaire-data

The questions are shown in Table 1.

**Table 1:** Questions in the questionnaire

Questions
Have you experienced dizziness during the past 30 days?
If yes, have you suffered from falls in relation to your dizziness?
If yes, have you sought help from a medical doctor for your dizziness?
Do you suffer from hearing loss?
Do you use specific medicine? (Antihypertensives, diuretics, anticoagulants, heart medication)
Do you suffer from allergies documented by your general practitioner?
Do you suffer from hypertension documented by your general practitioner?
Do you suffer from atherosclerosis, documented by your general practitioner?
How would you rate your health (‘very poor’, ‘poor’, ‘moderate’, ‘good’, ‘excellent’)?

### Physical measurements

Height, weight, creatinine, eGFR, sodium, potassium, hemoglobin, cholesterol value, and systolic and diastolic blood pressure were obtained.

### Sociodemographic data

Age in years, gender, marital status (married, separated/divorced, widowed, single), educational level (less than high school, high school, a bachelor’s degree, advanced degree, other), occupation (unemployed, out of the labor market, in labor, studying or training, work in the home, other), and the number of individuals in the household.

### Statistical analyses

All data management and statistical analyses were performed using R, version 4.1.1. Logistic regressions were applied to test for bivariate associations. For ordinal data, we applied the Cochran-Armitage test for trend.

A multivariate binary logistic regression model was fitted to explain binary variables from several quantitative and qualitative covariates. For the logistic regression, the participants answering ‘do not know’ to dizziness were removed. A Hosmer–Lemeshow test was then performed to check for the goodness of fit. Odds-ratio (OR) estimates, 95%-confidence intervals (CI), and p-values were derived from the logistic regression models.

### Ethics and dissemination

Region Zealand’s Ethical Committee on Health Research has approved LOFUS (Reg: SJ-421). All data storage and management for this study were approved by the Regional Data Protection Agency of Zealand (REG-065–2021 & REG-24–2015), and the use of data was accepted by the LOFUS steering committee. LOFUS is registered on http://www.clinicaltrials.gov (NCT02482896).

## Results

In total, 11,057 individuals above 50 years of age attended the LOFUS study. Of the 11,057 individuals, 10,092 answered the questions regarding dizziness and were eligible for this study. 5279 (52%) were female, and 4813 (48%) were male. The mean age was 64.7 (range: 50.1 to 96.6), and 65.7 (range: 50.0 to 95.6) for females and males, respectively. Of the 10,092 individuals included in this study, almost 20% had experienced dizziness during the past 30 days. For baseline characteristics, see Table 2.

**Table 2:** The demographics of the participants

Variables	Male (*N = *4813)	Female (*N = *5279)	Overall [10, 092]
Sex (percentage)	47.7%	52.3%	100%
Age			
Mean (SD)	65.7 (8.96)	64.7 (8.88)	65.2 (8.93)
Median [min, max]	65.6 [50.0, 95.6]	64.4 [50.0, 96.6]	64.9 [50.0, 96.6]
Age groups			
50–59 years old	1452 (30.2%)	1802 (34.1%)	3254 (32.2%)
60–69 years old	1766 (36.7%)	1940 (36.7%)	3706 (36.7%)
70–79 years old	1297 (26.9%)	1263 (23.9%)	2560 (25.4%)
> 80 years old	298 (6.2%)	274 (5.2%)	572 (5.7%)
BMI			
Mean (SD)	28.0 (4.39)	27.1 (5.30)	27.5 (4.91)
Median [Min, Max]	27.4 [16.5, 50.4]	26.1 [12.6, 54.0]	26.8 [12.6, 54.0]
Missing	129 (2.7%)	98 (1.9%)	227 (2.2%)
Civil status			
Widow/widower	231 (4.8%)	564 (10.7%)	795 (7.9%)
Married/Registered partnership	3646 (75.8%)	3696 (70.0%)	7342 (72.8%)
Divorced/separated	377 (7.8%)	489 (9.3%)	866 (8.6%)
Unmarried	523 (10.9%)	478 (9.1%)	1001 (9.9%)
Occupational position			
Old-age pensioner	2152 (44.7%)	2297 (43.5%)	4449 (44.1%)
Other	89 (1.8%)	138 (2.6%)	227 (2.2%)
Unemployed	53 (1.1%)	69 (1.3%)	122 (1.2%)
Employee or self-employed	2128 (44.2%)	2072 (39.2%)	4200 (41.6%)
Other forms of retirement	345 (7.2%)	592 (11.2%)	937 (9.3%)
Long-term sickness	10 (0.2%)	9 (0.2%)	19 (0.2%)
Student or apprentice	4 (0.1%)	10 (0.2%)	14 (0.1%)
Missing	32 (0.7%)	92 (1.7%)	124 (1.2%)
Dizziness			
Dizzy	817 (17.0%)	1147 (21.7%)	1964 (19.5%)
Not dizzy	3773 (78.4%)	3821 (72.4%)	7594 (75.2%)
Do not know	223 (4.6%)	311 (5.9%)	534 (5.3%)

### Gender effect

In total, 22% of the females reported dizziness compared with 17% of the males (Table 2). Dizziness was significantly more frequent in females than in males in all age groups. In the logistic regression model, a statistically significant difference in odds of dizziness between the female and male sex was found (OR = 1.52, 95% CI [1.34, 1.74]), see Table 3.

**Table 3:** Multivariate logistic regression with dizziness as response-variable

Variables	OR	95% CI	*p* value
Sex			
Male	Reference		
Female	1.52	1.34, 1.74	< 0.001
Age-groups			
50–59 years old	1.20	1.02, 1.42	0.031
60–69 years old	Reference		
70–79 years old	1.19	1.01, 1.41	0.034
> 80 years old	1.67	1.30, 2.15	< 0.001
Self-perceived health			
Very poor	3.62	1.75, 7.93	< 0.001
Poor	2.15	1.71, 2.72	< 0.001
Moderate	Reference		
Good	0.36	0.32, 0.40	< 0.001
Excellent	0.14	0.10, 0.18	< 0.001
Hearing loss			
No hearing loss	Reference		
Mild hearing loss	1.33	1.15, 1.53	< 0.001
Moderate hearing loss	1.63	1.37, 1.94	< 0.001
Severe hearing loss	2.40	1.77, 3.26	< 0.001
Do not know	1.34	1.12, 1.59	0.001
Use of antihypertensives			
No antihypertensives	Reference		
Antihypertensives	1.15	1.01, 1.30	0.031
Occupational position			
Employee or self-employed	Reference		
Old-age pensioner	1.20	0.99, 1.44	0.060
Other	1.34	0.94, 1.89	0.10
Unemployed	1.49	0.93, 2.35	0.089
Other forms of retirement	1.41	1.15, 1.73	< 0.001
Long-term sickness	3.00	1.05, 8.63	0.038
Student or apprentice	4.16	1.20, 13.1	0.017
Anticoagulants			
Yes	1.64	1.41, 1.89	< 0.001
No	Reference		
Hemoglobin			
	0.91	0.83, 0.99	0.026

### Age effect

The prevalence of dizziness increased with increasing age, being approximately 1.75 times more frequent in > 80 years of age compared with 50–59 years of age. When divided into age groups, a statistically significant difference in odds of dizziness was found for the age range 70–79 years old (OR = 1.19, 95% CI [1.01, 1.41]) and > 80 years old (OR = 1.67, 95% CI [1.30, 2.15]).

### Occupation

A univariate analysis showed a statistically significant difference in dizziness between different categories of education (Table 4). When the occupational position was tested in the multivariate logistic regression, only “other forms of retirement”, “long-term sickness” and “student/apprentice” were statistically significant. However, the number of participants answering “student/apprentice” and “long-term sickness” were very small (Table 3).

**Table 4:** Univariate analysis of dizziness

Variables	Not dizzy (*N = *7594)	Dizzy (*N = *1964)	Count in total	Univariate analysis	
				OR [95% CI]	*p* value
Sex					
Male	3773 (49.7%)	817 (41.6%)	4590	1.39 [1.25, 1.53]	< 0.001
Female	3821 (50.3%)	1147 (58.4%)	4968	Reference	
Age groups					
50–59 years old	2515 (33.1%)	586 (29.8%)	3101	1.04 [0.92, 1.17]	0.564
60–69 years old	2871 (37.8%)	645 (32.8%)	3516	Reference	
70–79 years old	1856 (24.4%)	560 (28.5%)	2416	1.34 [1.18, 1.53]	< 0.001
> 80 years old	352 (4.64%)	173 (8.81%)	525	2.19 [1.79, 2.67]	< 0.001
Self-perceived health					
Very poor	14 (0.185%)	28 (1.43%)	42	3.91 [2.05, 7.47]	< 0.001
Poor	188 (2.48%)	224 (11.4%)	412	2.33 [1.89, 2.88]	< 0.001
Moderate	1757 (23.2%)	898 (45.9%)	2655	Reference	
Good	4567 (60.3%)	748 (38.2%)	5315	0.32 [0.29, 0.36]	< 0.001
Excellent	1049 (13.8%)	60 (3.06%)	1109	0.11 [0.09, 0.15]	< 0.001
Hearing loss					
None	3812 (50.4%)	767 (39.3%)	4579	Reference	
Mild	1812 (24.0%)	495 (25.4%)	2307	1.36 [1.20, 1.54]	< 0.001
Moderate	861 (11.4%)	326 (16.7%)	1187	1.88 [1.62, 2.18]	< 0.001
Severe	149 (1.97%)	101 (5.17%)	250	3.37 [2.59, 4.39]	< 0.001
Do not know	924 (12.2%)	263 (13.5%)	1187	1.41 [1.21, 1.66]	< 0.001
Use of diuretics					
Diuretics	762 (10.3%)	356 (19.0%)	1118	2.04 [1.78, 2.34]	< 0.001
No diuretics	6609 (89.7%)	1513 (81.0%)	8122	Reference	
Use of antihypertensives					
Yes	5028 (67.3%)	1041 (54.6%)	6069	1.71 [1.54, 1.89]	< 0.001
No	2445 (32.7%)	864 (45.4%)	3309	Reference	
Occupational position					
Employee or self-employed	3409 (45.3%)	606 (31.6%)	4015	Reference	
Old-age pensioner	3237 (43.0%)	958 (50.0%)	4195	1.66 [1.49, 1.86]	< 0.001
Other	150 (1.99%)	60 (3.13%)	210	2.25 [1.65, 3.07]	< 0.001
Unemployed	85 (1.13%)	32 (1.67%)	117	2.12 [1.40, 3.21]	< 0.001
Other forms of retirement	634 (8.42%)	246 (12.8%)	880	2.18 [1.84, 2.59]	< 0.001
Long-term sickness	9 (0.119%)	9 (0.470%)	18	5.63 [2.22, 14.23]	< 0.001
Student or apprentice	8 (0.106%)	5 (0.261%)	13	3.52 [1.15, 10.78]	0.028
Anticoagulants					
Yes				2.36 [2.10, 2.65]	< 0.001
No				Reference	
Hemoglobin					
Mean (SD)	8.82 (0.699)	8.69 (0.740)		0.78 [0.72, 0.83]	< 0.001

### Antihypertensive and diuretic medicine

Dizziness was significantly more frequent in participants being treated with diuretics or antihypertensive medications (Table 4). In the logistic regression model, the use of antihypertensive medication was statistically significant (OR = 1.15, 95% CI [1.01, 1.30]. As diuretic medicine was included in the category of antihypertensive medicine, the use of diuretic medicine was not tested separately in logistic regression.

### Hearing loss

Of 1964 participants suffering from dizziness in the past 30 days, 40% also reported some degree of hearing loss (mild, moderate, or severe). Males reported more frequent hearing loss. The prevalence of hearing loss with dizziness is seen in Table 4. Logistic regression revealed a statistically significant increase in odds of dizziness between individuals with hearing loss and without hearing loss (mild hearing loss: OR = 1.33 [95% CI 1.15, 1.53], moderate hearing loss: OR = 1.63 [95% CI 1.37, 1.94], and severe hearing loss: OR = 2.40 [95% CI 1.77, 3.26]) when adjusting for covariates and suspected confounders such as age.

### Self-perception of health

In logistic regression, a significant difference in odds of dizziness was seen between different degrees of self-perceived health. Poor self-perceived health and very poor self-perceived health revealed odds ratios of 2.15 (95% CI 1.71, 2.72), and 3.62 (95% CI 1.75, 7.93), respectively. Oppositely, good- and excellent self-perceived health revealed odds ratios of OR = 0.36, 95% CI (0.32, 0.40), and OR = 0.14, 95% CI (0.10, 0.18).

### Experiencing falls and seeking treatment due to dizziness

Of the 1964 participants answering “yes” to dizziness during the past 30 days, 1951 participants answered the question about the occurrence of falls in relation to their dizziness (Table 5). 24% of the women suffering from dizziness in the past 30 days had experienced falls compared to 21% of men. An increase in the occurrence of falls was seen with increasing age in both sexes. In logistic regression, the best predictors of falls were very poor, poor, and good self-perceived health with an OR = 3.09, 95% CI (1.27–7.57), OR = 1.58, 95% CI (1.13, 2.21), and OR = 0.52, 95%- CI (0.4–0.67), respectively. Furthermore, increasing age also increased the odds ratio of falls, see Table 6.

**Table 5:** Characteristics of the dizzy and non-dizzy participants

	Dizzy		Not dizzy		Do not know	
	Male (*N = *817)	Female (*N = *1147)	Male (*N = *3773)	Female *N = *3821)	Male (*N = *223)	Female (*N = *311)
Age-groups						
50–59 years	203 (24.8%)	383 (33.4%)	1190 (31.5%)	1325 (34.7%)	59 (26.5%)	94 (30.2%)
60–69 years	270 (33.0%)	375 (32.7%)	1420 (37.6%)	1451 (38.0%)	76 (34.1%)	114 (36.7%)
70–79 years	271 (33.2%)	289 (25.2%)	965 (25.6%)	891 (23.3%)	61 (27.4%)	83 (26.7%)
> 80 years old	73 (8.9%)	100 (8.7%)	198 (5.2%)	154 (4.0%)	27 (12.1%)	20 (6.4%)
Self-perceived health						
Moderate	368 (45.0%)	530 (46.2%)	901 (23.9%)	856 (22.4%)	80 (35.9%)	132 (42.4%)
Very poor	12 (1.5%)	16 (1.4%)	9 (0.2%)	5 (0.1%)	1 (0.4%)	1 (0.3%)
Poor	98 (12.0%)	126 (11.0%)	89 (2.4%)	99 (2.6%)	21 (9.4%)	19 (6.1%)
Good	306 (37.5%)	442 (38.5%)	2248 (59.6%)	2319 (60.7%)	108 (48.4%)	144 (46.3%)
Excellent	28 (3.4%)	32 (2.8%)	514 (13.6%)	535 (14.0%)	13 (5.8%)	14 (4.5%)
Missing	5 (0.6%)	1 (0.1%)	12 (0.3%)	7 (0.2%)	0 (0%)	1 (0.3%)
Hearing loss						
No hearing loss	241 (29.5%)	526 (45.9%)	1551 (41.1%)	2261 (59.2%)	54 (24.2%)	143 (46.0%)
Mild hearing loss	252 (30.8%)	243 (21.2%)	1122 (29.7%)	690 (18.1%)	74 (33.2%)	54 (17.4%)
Moderate hearing loss	187 (22.9%)	139 (12.1%)	600 (15.9%)	261 (6.8%)	35 (15.7%)	27 (8.7%)
Severe hearing loss	52 (6.4%)	49 (4.3%)	97 (2.6%)	52 (1.4%)	20 (9.0%)	9 (2.9%)
Do not know	80 (9.8%)	183 (16.0%)	386 (10.2%)	538 (14.1%)	39 (17.5%)	76 (24.4%)
Missing	5 (0.6%)	7 (0.6%)	17 (0.5%)	19 (0.5%)	1 (0.4%)	2 (0.6%)
Treatment for dizziness						
Treatment	349 (42.7%)	494 (43.1%)				
No treatment	450 (55.1%)	621 (54.1%)				
Do not know	12 (1.5%)	27 (2.4%)				
Missing	6 (0.7%)	5 (0.4%)				
Falls due to dizziness						
Falls	175 (21.4%)	275 (24.0%)				
No falls	613 (75.0%)	813 (70.9%)				
Do not know	24 (2.9%)	51 (4.4%)				
Missing	5 (0.6%)	8 (0.7%)

**Table 6:** Multivariate logistic regression with falls as response-variable

Variables	OR	95% CI	*p* value
Sex			
Male	Reference		
Female	1.20	0.93, 1.54	0.2
Age-groups			
50–59 years old	0.89	0.66, 1.21	0.5
60–69 years old	Reference		
70–79 years old	1.41	1.06, 1.88	0.019
> 80 years old	1.64	1.06, 2.48	0.023
Self-perceived health			
Very poor	3.09	1.27, 7.57	0.012
Poor	1.58	1.13, 2.21	0.007
Moderate	Reference		
Good	0.52	0.4, 0.67	< 0.001
Excellent	0.53	0.24, 1.05	0.087
Use of antihypertensives			
No antihypertensives	Reference		
Antihypertensives	1.11	0.88, 1.40	0.4
Hemoglobin			
	0.89	0.76, 1.05	0.2

A total of 1,953 participants suffering from dizziness during the past 30 days answered the question about seeking treatment for their dizziness. 43% of both females and males stated that they had sought treatment for their dizziness (Table 5). Seeking treatment for dizziness increased with increasing age for men, however, the biggest increase for females was seen in the age group of 60–70 years, where almost 50% sought treatment for their dizziness.

Logistic regression for “seeking treatment for dizziness” as an outcome showed that the occurrence of falls due to dizziness was the best predictor of seeking treatment for dizziness with an OR = 3.21, 95% CI (2.53, 4.08), please see Table 7. Furthermore, good or excellent self-perceived health decreased the odds of seeking treatment (good self-perceived health OR = 0.8, 95% CI (0.65–0.99), excellent self-perceived health OR = 0.44, 95% CI (0.24–0.85).

**Table 7:** Multivariate logistic regression with seeking treatment for dizziness as response-variable

Variables	OR	95% CI	*p* value
Sex			
Male	Reference		
Female	1.03	0.83, 1.28	0.8
Falls due to dizziness			
No falls	Reference		
Falls due to dizziness	3.21	2.53, 4.08	< 0.001
Age-groups			
50–59 years old	Reference		
60–69 years old	1.35	1.05, 1.74	0.019
70–79 years old	1.14	0.87, 1.49	0.3
> 80 years old	1.27	0.86, 1.88	0.2
Self-perceived health			
Very poor	1.15	0.46, 2.96	0.8
Poor	1.41	1.01, 1.95	0.041
Moderate	Reference		
Good	0.8	0.65, 1.00	0.047
Excellent	0.44	0.24, 0.85	0.016
Use of antihypertensives			
No antihypertensives	Reference		
Antihypertensives	1.12	0.92, 1.38	0.3
Hemoglobin			
	0.93	0.81, 1.08	0.3

## Discussion

Dizziness is among the most common complaints in populations. Dizziness is associated with a significant degree of handicap, perception of low health, and occupational disability. Approximately one in five of the population in our study reported dizziness during the past month which is consistent with other studies reporting the monthly prevalence of dizziness [10, 11]. However, the mean age in our study was 65 years, which is not comparable to the mean age in other studies. In the United Kingdom, Yardley et al. found that 23% experienced dizziness during the past month (mean-age for females was 38.7) [10]. In Norway, Wiltink et al. found that 15.8% experienced dizziness during the past month in a population with a mean age of 48.8 [11].

The prevalence of dizziness increased with age, and there was a female predominance in our data. These findings are supported by several other studies [12, 13]. If a dizzy individual seeks treatment from a medical doctor, it may indicate that dizziness is severe and possibly influences the quality of life. This was the case in 43% of the participants in our study.

We demonstrated that a good predictor for seeking treatment for dizziness was the occurrence of falls in relation to dizziness. This was not unexpected. However, if many dizzy individuals only seek help after experiencing falls in relation to dizziness, it may be difficult to prevent falls. Various reasons could explain why individuals suffering from dizziness do not seek treatment from a medical doctor. One reason among others could be that the individual does not feel that the dizziness complaint is being taken seriously by their general practitioner or other health professionals. This could be due to the fact that many find it difficult to diagnose and treat dizziness but also that comorbidities often are present. Another reason could be relatives who think that it is normal to get dizzy with increasing age and therefore do not respond to the complaint. One might argue that an invitation for vestibular rehabilitation could help at least some of the patients as well as society by preventing fractures, especially hip fractures, and other events to happen.

We found that hearing loss and dizziness are often associated. This is probably mainly due to age-related factors like neurodegenerative processes in the elderly. The association is not surprising.

As regards subjective general health, we found that 13% experienced a “very poor” or “poor” subjective general health with a statistically significant difference between the dizzy and non-dizzy groups. This is congruent with data from Sweden where 18% reported “very poor” or “poor” health [14]. However, the data from Sweden reporting “very poor” or “poor” health might be a bit higher compared to our data because included patients were 79 years of age compared to our sample with a mean age of 65 years. Data from Brazil conclude the same: that self-perception of health was rated lower in dizzy- than in non-dizzy individuals [13].

As dizziness as a symptom is non-specific and may arise from several organ systems, it is not surprising that a correlation was found with general health, use of medication, and hearing loss. Consequently, the findings do not allow any specific conclusions on the contributing factors to dizziness and falls. Furthermore, the directionality of causalities may be in two or multiple ways.

## Limitations and strengths

This is a cross-sectional questionnaire survey that only allows for possible associations but does not allow for causative explanations. In addition, participants who answered this questionnaire may be subject to bias by indication. A study investigating the degree of bias in LOFUS with logistic regression found that odds ratios for participation increased with income, education level, and employment status, among married individuals, Danish citizens, middle-aged individuals (aged 50–69 years), and female gender. Briefly, significantly higher participation of women and individuals of higher socioeconomic status was found, implying that people of lower socioeconomic status were underrepresented in LOFUS, in line with other cohort studies [15].

The strength is that the survey includes more than 10,000 participants in the age group for dizziness. Also, participants were not asked questions concerning possible associated items in the same section as dizziness. This makes it quite speculative that participants were skewed in their answers.

## Conclusions

The prevalence of dizziness in citizens aged 50 years and above and participating in the Lolland-Falster Health Study was 20% which is in line with other similar studies. Citizens suffering from dizziness were negatively associated with self-perception of health also after adjusting for other comorbidities. 43% of dizzy patients sought treatment for their dizziness. 23% of the dizzy citizens experienced falls due to dizziness. The occurrence of falls was an indicator of seeking medical examination. Identification of dizziness, as well as treatment such as vestibular rehabilitation, is important to prevent falls from happening in the nearby future.

## Data Availability

The data that support the findings of this study are available on request from the corresponding author, CG. The data are not publicly available as it contains information that could compromise the privacy of research participants.
